# Molecular surveillance of chloroquine resistance in *Plasmodium vivax* isolates from malaria cases in Yunnan Province of China using *pvcrt-o* gene polymorphisms

**DOI:** 10.1186/s12936-023-04776-z

**Published:** 2023-11-08

**Authors:** Hongyun Ding, Ying Dong, Yan Deng, Yanchun Xu, Yan Liu, Jing Wu, Mengni Chen, Canglin Zhang, Li Liu, Yingkun Lin

**Affiliations:** 1 Faculty of Life Science and Technology, Kunming University of Science and Technology, Kunming, 650500 China; 2 Yunnan Provincial Key Laboratory of Vector-borne Diseases Control and Research, Yunnan International Joint Laboratory of Tropical Infectious Diseases, Yunnan Institute of Parasitic Diseases Control, Pu’er, 665000 China; 3 Center for Disease Control and Prevention, Dehong, 678499 China

**Keywords:** Monitoring, Yunnan, *Plasmodium vivax*, Infected strains, Chloroquine, Drug resistance, Molecular markers, Chloroquine transporter protein

## Abstract

**Background:**

The efficacy of chloroquine treatment for vivax malaria has been rarely evaluated due to a lack of an appropriate testing method. The objective of this study was to conduct molecular monitoring of chloroquine resistance in *Plasmodium vivax* strains from vivax malaria patients in Yunnan Province, focusing on the analysis of polymorphism in the *P. vivax* chloroquine resistance transporter protein orthologous gene (*pvcrt-o*).

**Methods:**

In accordance with the principles of a cohort study, blood samples were collected from malaria cases diagnosed with a *P. vivax* mono-infection in Yunnan Province from 2020 to 2022. Segmental PCR was used to amplify the whole *pvcrt-o* gene in the blood samples and their products were subsequently sequenced. The sequencing data were arranged to obtain the full coding DNA sequence (CDS) as well as the gene’s promoter region sequences. The CDSs were aligned with the reference sequence (XM_001613407.1) of the *P. vivax* SalI isolate to identify the mutant loci.

**Results:**

From a total of 375 blood samples taken from vivax malaria cases, 272 both whole gene CDSs (1272–1275 bp) and promoter DNA sequences (707 bp) of *pvcrt-o* gene were obtained. Among the whole CDSs, there were 7 single nucleotide polymorphic sites in which c.7 A>G was the minor allele frequency (MAF) site with 4.4% (12/272) detection rate. The mutation detection rate showed a significant decrease from 9.8% (10/102) in 2020 to 1.1% (1/92) in 2021 and 1.3% (1/78) in 2022, indicating statistical significance (*χ*^2^ = 11.256, *P* < 0.05). Among the identified 12 haplotypes, the majority of which were wild type (75.7%; 206/272). These four mutant haplotypes (Hap_3, Hap_5, Hap_9, and Hap_10) were classified as “K10 insertion type” and accounted for 12.1% (33/272). The detection rate of Hap_3 increased from 1.0% (1/102) in 2020 to 13.0% (12/92) in 2021 and 14.1% (11/78) in 2022, indicating statistical significance. A total of 23.8% (65/272) of the samples exhibited 14 bp (bp) deletions in the promoter region, occurring most frequently in the wild type haplotype (Hap_1) samples at a rate of 28.6% (59/206).

**Conclusions:**

In recent years in Yunnan Province, a notable proportion of vivax malaria patients are infected by *P. vivax* strains with a “K10 insertion” and partial sequence deletions in the promoter region of the *pvcrt-o* gene, necessitating vigilance.

**Supplementary Information:**

The online version contains supplementary material available at 10.1186/s12936-023-04776-z.

## Background

Chloroquine (CQ), an anti-malarial drug that kills intraerythrocytic *Plasmodium* parasites, has long been the preferred treatment for controlling and eliminating the clinical symptoms of malaria, since it was first synthesized in 1934 [1, 2], and it has been extensively used in Yunnan Province since 1960 [3]. However, in 1973 [4, 5] and 1974 [6], reports from China’s Yunnan and Hainan provinces began to appear which suggested the emergence of severe chloroquine-resistant malaria. Subsequently, CQ-resistant strains continued to spread, while CQ resistance increased [5].

Based on available data, the first instances of CQ treatment failure in vivax malaria cases were observed in Papua New Guinea (1989) [7] and Indonesia (1991) [8]. Later, other countries with endemic vivax malaria, including Myanmar [9], Vietnam [10], Turkey [11], Pakistan [12], and Afghanistan [12], also reported appearing CQ resistance in *Plasmodium vivax* strains. In China, the first case of CQ treatment failure was reported in 1985 [13], and in 1996, four vivax malaria cases of CQ treatment failure were discovered again in Yunnan Province [14].

Despite an extensive focus on addressing CQ resistance in *Plasmodium falciparum* in Yunnan Province [15–17], the evaluation of the efficacy of CQ treatment vivax malaria in the region has remained insufficient to effectively manage the persistent epidemic burden. Since 1958, Yunnan has consistently employed CQ (total dose of 1200 mg, administered orally over 3 days) as the first-line anti-malarial drug for treatment of the vivax malaria clinical episodes [18–21]. In the past 40 years alone, incomplete statistics indicate that over 300,000 individuals have received CQ treatment [22–27]. Although Yunnan Province reported the last indigenous vivax malaria case in China in April 2016 [28], a total of 1371 imported vivax malaria cases had been reported up until the end of 2022 [22, 28, 29]. These imported cases originated in Myanmar, Nigeria, the Democratic Republic of Congo, Angola, and Cameroon, and primarily impacted southeast Asian countries particularly Myanmar, which experienced the highest number of imported cases [22, 28, 29]. While the investigation of vivax malaria transmission sources can achieve precision, the assessment of treatment efficacy for vivax malaria cases in Yunnan Province remains inadequate due to the limited practicality of the traditional detection methods.

The methods for testing *P. vivax* resistance to an anti-malarial drug, which include both in vivo and in vitro and molecular marker detection, were always introduced from assessing drug resistance in *P. falciparum* [30, 31]. However, the in vivo accuracy of evaluating the efficacy of anti-malarial drug already faces a challenge due to the difficulty to distinguishing between treatment failure and these infection events, such as recurrence or new infection [32, 33]. Additionally, the fact that *P. vivax* does not invade mature red blood cells, makes it difficult to establish a continuous in vitro culture system [34, 35]. Consequently, the in vitro testing methods commonly used for monitoring drug sensitivity in *P. falciparum* cannot be effectively applied to *P. vivax* [30, 32]. Moreover, there is additional challenges that the number of eligible volunteers for in vivo evaluation method has sharply decreased [36]. Given that the majority of vivax malaria cases harbour only low-density *P. vivax*, these low levels do not meet the necessary density threshold for conducting in vitro tests. Therefore, in 2018 the World Health Organization (WHO) recommended the selective implementation of molecular marker monitoring to compensate for the unsystematic evaluation methods that have typically characterized anti-malarial drug efficacy studies [36].

However, the selection of molecular markers for drug resistance in *P. vivax* has been reliant on research findings derived from *P. falciparum* studies. This is partly due to the slow progress of genomics research specifically targeting *P. vivax*, resulting in research data with limited practical application value [37]. On the other hand, the confirmation of orthologous genes between *P. falciparum* and *P. vivax* [38–40] has led to valuable insights. Not only has this discovery provided a reference template for the genome assembly of *P. vivax*, but it has also made it possible to transfer techniques and analytical tools previously developed for the study of *P. falciparum* to *P. vivax* [40]. Within the orthologous genes shared between *P. falciparum* and *P. vivax*, the *P. vivax* chloroquine resistance transporter protein orthologous gene (*pvcrt-o*) was considered as a counterpart to the chloroquine resistance molecular marker found in the *P. falciparum* chloroquine resistance transporter protein gene (*pfcrt*) [32, 41, 42]. Furthermore, the role of *pfcrt* as a molecular marker for chloroquine resistance has undergone verification across various levels.

The *P. falciparum* chloroquine resistance transport protein (*Pf*CRT), encoded by the *pfcrt* gene, is a membrane protein component of *P. falciparum*’s digestive vacuole. *Pf*CRT plays a crucial role in mediating the accumulation of CQ within *P. falciparum* by exhibiting transmembrane characteristics that facilitate CQ transport [43]. Pairwise studies conducted by Nomura et al. [41] demonstrated that mutations in the coding region of the *pfcrt* gene—particularly, missense mutations at amino acid codons 72–76 in exon 2 (C72S, M74I, N75E, and K76T) [41] significantly affect the function of *Pf*CRT in *P. falciparum*. Among these mutations, the K76T mutation is particularly notable as it causes a reduction in the hydrophobic structure of *Pf*CRT and impairs the binding of CQ in the digestive vacuole [44, 45]. Therefore, the K76T mutation has emerged as a reliable molecular biomarker for detecting CQ resistance in *P. falciparum* infections [46–48].

To evaluate *pvcrt-o* as a molecular marker for CQ resistance in *P. vivax*, Sá et al. introduced *pvcrt-o* into a CQ-sensitive *P. falciparum* model, which resulted in a 2.2-fold increase in its tolerance to CQ. This result indirectly implies the likely effects of *pvcrt-o* on CQ resistance in *P. vivax* infections [49]. In another study, Fernández-Becerra et al. compared the transcript levels of two transporter proteins, *Pf*CRT and *Pf*MDR1, which are believed to be involved in the formation of CQ resistance in *P. vivax*. The researchers found that the expression levels of the *pvcrt-o* and *pvmdr1* genes in severe *P. vivax* infections were 21.9-fold and 2.9-fold higher, respectively, than in mild cases. This result indicates a possible association between increased gene expression levels and clinical severity and highlights the potential role of these genes—particularly *pvcrt-o*—as molecular markers for drug resistance in *P. vivax* [50]. Additionally, Suwanarusk et al. [51]. suggested that the occurrence of a K10 insertion mutation in the *pvcrt-o* gene of *P. vivax* was significantly associated with a higher IC_50_ value for CQ treatment in *P. vivax* infection [51].

To improve on the assessing capabilities of CQ treatment efficacy for *P. vivax* infection in Yunnan Province and to facilitate routine monitoring of anti-malarial drug efficacy [36], the molecular marker *pvcrt-o* will be included in the research field of Yunnan Province. This study presents a comprehensive cohort sequencing analysis to elucidate the polymorphic mutations in the full *pvcrt-o* gene within vivax malaria cases’ strains collected from Yunnan Province from 2020 to 2022. The findings are detailed below.

## Methods

### Study subjects and blood samples

This study utilized a cohort study design and included all vivax malaria cases diagnosed and confirmed in Yunnan Province from 2020 to 2022. These cases were also included in the “China Disease Surveillance Information Reporting and Management System.” Each case was initially diagnosed by county laboratories across Yunnan Province and later confirmed by the Yunnan Province Malaria Diagnosis Referent Laboratory (YPMDRL). Confirmation methods involved both blood smear examination using optical microscopy as well as genetic testing to ensure the presence of a mono-*P. vivax* infection. Further details about the primer sequences, reaction conditions, and reaction system used for *Plasmodium* genetic testing can be found in Additional file 1. Gender, age, initial diagnosis location, and the source of infection for each vivax malaria case were also recorded in compliance with the “China Disease Surveillance Information Reporting and Management System.”

Peripheral venous blood samples were collected from all acute episodes of vivax malaria cases and dried blood spots (DBS) onto filter paper for genetic sequencing analysis.

### Extraction of *Plasmodium* genome DNA and PCR amplification of the *pvcrt-o* gene

Three 5 mm-diameter dried blood drops (DBDs) were applied to extract *Plasmodium* genome DNA according to the instructions provided by the QIAgen DNA Mini Kit (DNA Mini Kit, QIAamp, Germany). The extracted DNA was stored at − 20 °C for future use.

To obtain the full coding DNA sequence (CDS) of the *pvcrt-o* gene, PCR amplification was applied to specific segments which were subsequently sequenced. The chromosomal reference sequence (ID: NC_009906.1) of the *P. vivax* SalI isolate was utilized as a template for the design of primers that would allow to amplify the whole *pvcrt-o* gene and its promoter region (Table 1).

**Table 1:** The primer names and the sequences, target fragment lengths of PCR amplification for the pvcrt-o gene in P. vivax strains

Fragments	Primer names	Primer sequences (5′→3′)	Product length (bp)	Amplification interval
F1	Vcrt-o-PR-F1	TTTTTATTTCCTCACGTGCTAAGTG	2223	329,998–332,220
	Vcrt-o-1R1	TGAATGCGCCGACGTAATT		
F2	Vcrt-o-5F1	GTTATCATCGCGTTTATAGGTAATT	2139	331,778–333,916
	Vcrt-o-9R1	GGTGTAGGTCATGGTTGAGAAT		
F3	Vcrt-o-11F1	GCGACAATTTAATCACCAGCTT	1127	333,685–334,811
	Vcrt-o-11R1	TTGGCTCATGAGCGGGTTAG		

Specifically, Fragment 1 (F1, 329,998–332,220 nt) covered the promoter, exons 1–7, and introns 1–6; Fragment 2 (F2, 331,778–333,916 nt) covered exons 8–10 and introns 7–9; Fragment 3 (F3, 333,685–334,811 nt) covered exons 11–14 and introns 10–13. Detailed information about the PCR amplification primers for these three fragments can be found in Additional file 2.

The preparation for all three PCR reactions consisted of 2.0 µL of DNA template, 14.0 µL of PCR mixing system with 2× Taq, 0.7 µL each of upstream and downstream primers (10 µmol/L), and a volume adjusted to 25.0 µL with ddH2O. The reaction conditions were as follows: pre-denaturation at 95 °C for four minutes; 35 cycles of amplification followed by the procedure including denaturation at 95 °C for 45 s; annealing at 55–61 °C for 45 s; extension at 72 °C for 120–180 s. The PCR amplification was terminated at 72 °C for 10 min. The resulting PCR amplification products were visualized using 1.2% agarose gel electrophoresis, with the lengths of the three PCR amplification products estimated at 2200 bp, 2200 bp and 1200 bp. The positive products were then sent to Guangzhou Tianyi Huiyuan Gene Technology Co. Ltd. for Sanger Paired-End Sequencing.

### Gene polymorphism analysis

The sequencing data obtained from the F1 amplification products included the *pvcrt-o* promoter region and exons 1–7, spanning 1–707 bp. Similarly, the sequencing data from the F2 and F3 amplification products provided the sequences for exons 8–10 and exons 11–14, respectively. Each exon was then concatenated in the 5′→3′ direction to form the complete CDS of the *pvcrt-o* gene. Comparisons were made to verify the accuracy and relevance of the compiled promoter sequence and CDS. The promoter sequence of each sample was compared with the reference sequence (ID: NC_009906.1) and the CDS was compared with the reference mRNA sequence of the *pvcrt-o* gene (ID: XM_001613407.1). If both the coverage (Query cover) and the consistency of sequence alignment (Per. Ident) are greater than 98%, it indicates that the compiled sequences are correct and relevant to the *pvcrt-o* gene.

The samples included in the bioinformatics analysis provided both the full CDS and the promoter DNA sequence of *pvcrt-o*. Using MEGA 5.04 software, a file was generated to align the promoter sequence and the CDS, the latter of which was used to deduce the corresponding amino acid sequence. DnaSP 6.11.01 software was employed to identify haplotypes and single nucleotide polymorphism (SNP) sites within the CDS region of *pvcrt-o*. The software also determined the mutation types, distinguishing between synonymous and non-synonymous mutations. To assess the presence of multiple mutations in the CDS sequence, nucleotide diversity (π), expected heterozygosity (He), and Ka/Ks ratio was calculated. Using Network 10.0 software, intermediate network evolutionary diagrams were constructed to visualize the relationships between various haplotypes.

For all SNP sites, the base substitution forms can be verified and confirmed using the corresponding sequencing files (“.ab1” files). The Ka/Ks ratio serves as an indicator of natural selection within a given population. A ratio greater than 1, equal to 1, or less than 1 indicates positive selection, neutral selection, or negative selection, respectively [52].

### The transmembrane regions of *Pv*CRT in the food vacuole

The 3D structure of the *P. vivax* chloroquine resistance transporter protein (*Pv*CRT) within *Plasmodium*’s food vacuole membrane was predicted using the amino acid reference sequence (ID: XP_001613457.1) provided by the SWISS-MODEL online software (https://swissmodel.expasy.org/interactive). The α-helical structures of the ten transmembrane regions have start and end positions that were observed to correspond to the following amino acid ranges: 58th–81th aa, 88th–108th aa, 127th–147th aa, 153th–170th aa, 181th–200th aa, 210th–229th aa, 242th–265th aa, 316th–336th aa, 341th–359th aa and 374th–394th aa (Additional file 3). The C-terminal and N-terminal structural domains of *Pv*CRT are located in the parasite cytoplasm [41].

### Statistical analysis

A polymorphism analysis database for the *pvcrt-o* gene was created using Microsoft Excel software. To analyse the data, IBM’s SPSS Statistics 21 software was employed, specifically by utilizing the Data and Descriptive Statistics module. A chi-square test (*χ*^2^) was conducted to investigate variations in the detection rates of SNPs and haplotypes across different years. The significance level for the test was set at 0.05.

## Results

### Sample information and PCR amplification

In this study, mutations in *pvcrt-o* gene were detected using peripheral blood samples collected from 375 vivax malaria cases diagnosed in Yunnan Province, China, from January 2020 to December 2022. The study population consisted primarily of males (77.9%, 292/375) and adults aged from 16 to 60 years old (76.8%, 288/375). Most of the infections were acquired in Myanmar (99.2%, 372/375), with only one case each originating in Africa, Thailand, and Pakistan (Table 2).

**Table 2:** Information on vivax malaria cases diagnosed in Yunnan Province from January 2020 to December 2022

	Total No. (P, %)	2020 No. (P, %)	2021 No. (P, %)	2022 No. (P, %)
Total	375 (100.0)	154 (41.1)	126 (33.6)	95 (25.3)
1. Gender				
Male	292 (77.9)	122 (41.8)	100 (34.2)	70 (24.0)
Female	83 (22.1)	32 (38.6)	26 (31.3)	25 (30.1)
2. Age				
0–20	70 (18.7)	25 (35.7)	23 (32.9)	22 (31.4)
21–60	288 (76.8)	125 (43.4)	97 (33.7)	66 (22.9)
>60	17 (4.5)	4 (23.5)	6 (35.3)	7 (41.2)
3. Source of infection				
Myanmar	372 (99.2)	153 (41.1)	125 (33.6)	94 (25.3)
Africa	1 (0.3)	0	1 (100.0)	0
Thailand	1 (0.3)	0	0	1 (100.0)
Pakistan	1 (0.3)	1 (100.0)	0	0

*P* Proportion

The distribution of the 375 vivax malaria cases is as follows: Dehong accounts for 274 cases (73.1%, 274/375), followed by Baoshan with 45 cases (12.0%, 45/375), Lincang with 32 cases (8.5%, 32/375), and Kunming with 12 cases (3.2%, 12/375). Dali and Honghe each reported 4 cases (1.1%, 4/375), Xishuangbanna identified 2 cases (0.5%, 2/375), and both Chuxiong and Qujing registered 1 case each (0.3%, 1/375). (Additional file 4).

**Fig. 1:**
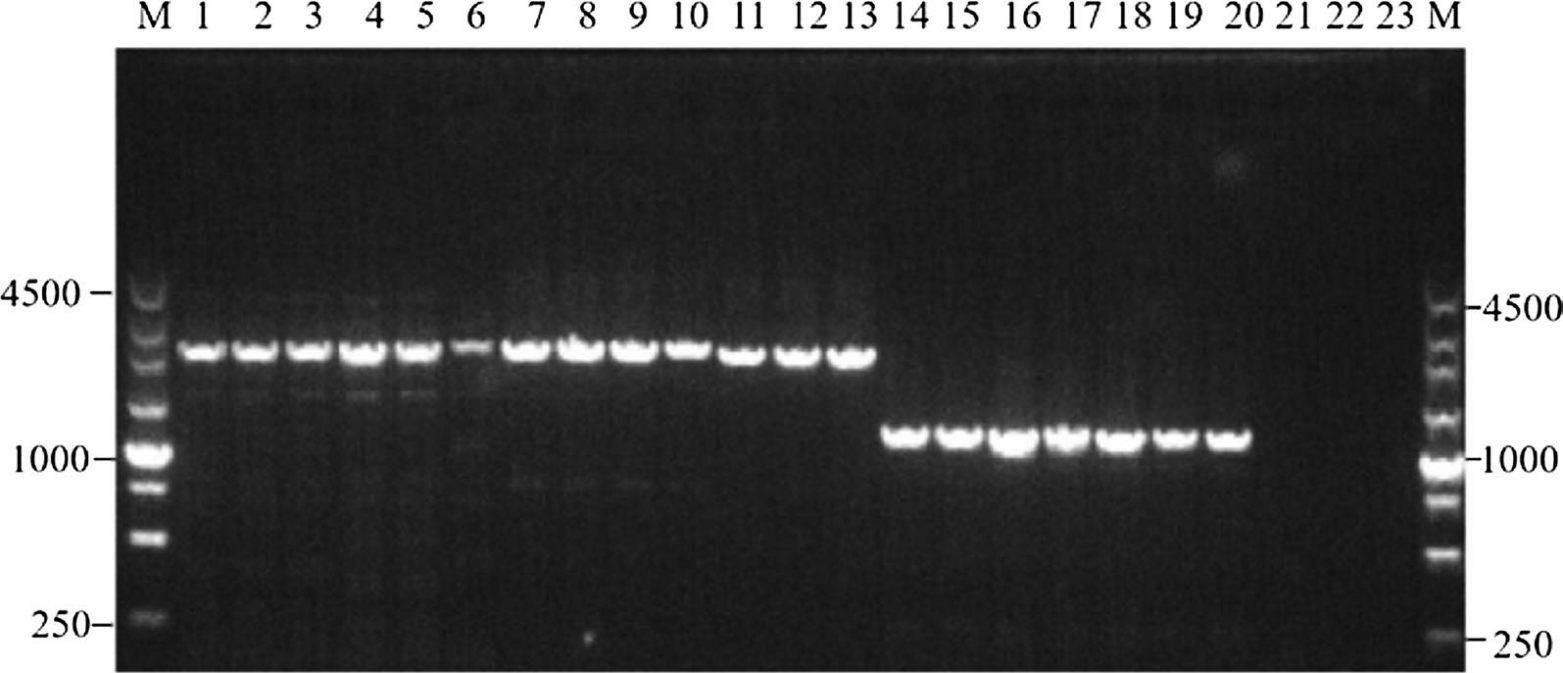
Electrophoresis of the PCR amplification products of the *pvcrt-o* gene in *P. vivax* strains from vivax malaria cases in Yunnan Province. (1) M: DNA marker; (2) 21, 22, 23: negative control of the primary PCR; 1, 2, 3, 4, 5, 6: amplification products of fragment F1 for the PCR; 7, 8, 9, 10, 11, 12, 13: amplification products of fragment F2 for the PCR; 14, 15, 16, 17, 18, 19, 20: amplification products of fragment F3 for the PCR

Out of the collected 375 vivax malaria cases blood samples, 304 samples were successfully amplified to obtain the target fragment with 2300 bp, 2250 bp, and 1200 bp electrophoresis bands following segmental PCR amplification of the *pvcrt-o* gene (Fig. 1). The success rate of the amplification was 81.1% (304/375).

### Nucleotide diversity of *pvcrt-o* gene CDSs

Upon the organization and assembly of the sequencing results from the afore mentioned 304 PCR product groups, 272 samples were successfully able to acquire both the complete CDS sequence (ranging from 1272 to 1275 bp) of the *pvcrt-o* gene and the DNA sequence of the promoter region. The consistency (Per. Ident) and coverage (Query cover) between these 272 CDSs (GenBank ID including: BankIt2619842: OR113083–OR113354 and will be held confidential until June 16 2024) and the reference sequence (ID: XM_001613407.1) were both greater than 98%. The nucleotide diversity index (π) and the Ka/Ks ratio were determined to be 0.00029 and 3.45011, respectively. CDSs were distributed across the years 2020, 2021, and 2022, accounting for 37.5% (102/272), 33.8% (92/272), and 28.7% (78/272) of the samples, respectively (Table 3). The nucleotide diversity index (π) and the Ka/Ks ratio for each year were as follows: 2020 (0.00045; 3.44978), 2021 (0.00015; 3.45026), and 2022 (0.00021; 3.45026).

**Table 3:** SNPs of the full-length CDS of *pvcrt-o* gene in *P. vivax* strains from vivax malaria cases

OD	Loci	AL	Coding	AAV	TMD	Frequency			
						No. of all CDSs (n = 272) No. (F, %)	2020 (n = 102) No. (F, %)	2021 (n = 92) No. (F, %)	2022 (n = 78) No. (F, %)
1	c.7	A>G	***A***TC/***G***TC	I3V	Outside	12 (4.4)	10 (3.7)	1 (0.4)	1 (0.4)
2	c.37	C>T	***C***CC/***T***CC	P13S	Outside	2 (0.7)	0	1 (0.4)	1 (0.4)
3	c.772	T>A	***T***GC/***A***GC	C258S	7	3 (1.1)	1 (0.4)	2 (0.7)	0
4	c.936	C>T	TG***C***/TG***T***	C312C	Inside	2 (0.7)	1 (0.4)	1 (0.4)	0
5	c.987	C>T	GA***C***/GA***T***	D329D	8	3 (1.1)	1 (0.4)	0	2 (0.7)
6	c.1032	C>G	AC***C***/AC***G***	T344T	9	4 (1.5)	3 (1.1)	1 (0.4)	0
7	c.1164	C>T	TC***C***/TC***T***	S388S	10	27 (9.9)	17 (6.3)	3 (1.1)	7 (2.6)

OD, AL, AAV, TMD, F are order, alleles, amino acid variation, transmembrane domain (inside and outside mean the inside and outside the digestive vesicle and frequency; the different Arabic numerals mean some transmembrane region), respectively

bolditalic values used to indicate that the mutant type base of the triplet code on the right where it is replaced from the original type on the left

The 272 CDSs had base substitutions at only seven sites, including c.7 and c.37 (Table 3, Additional file 5), but none of them were distributed in the coding region of amino acids 72th–76th. Among these CDSs, three non-synonymous mutations were concentrated in exons 9–14, while four synonymous mutations were located primarily in exons 1–7. Additionally, three base substitutions occurred at the first position of the codon, while four occurred at the third position (Table 3). c.7 A>G site mutation belongs to MAF (4.4%, 12/272), and the mutation detection rate at this site decreased from 9.8% (10/102) in 2020 to 1.1% (1/92) in 2021 and 1.3% (1/78) in 2022, a reduction that was statistically significant (*χ*^2^ = 11.256, *P* < 0.05) (Additional file 6) .

The number of detected SNP types in the CDSs for the years 2020, 2021, and 2022 were 6, 6, and 4, respectively. Specific mutations were observed at different frequencies within each year as follows: in 2020 and 2021, c.772T>A, c.936 C>T, and c.1032 C>G mutations were detected in 1.0% (1/102) and 2.2% (2/92), 1.0% (1/102) and 1.1% (1/92), and 2.9% (3/102) and 1.1% (1/92) of CDSs, respectively; c.37 C>T site mutations were detected in 1.1% (1/92) of the CDSs in 2021 and 1.3% (1/78) of the sequences in 2022; the c.987 C>T mutation was detected in 1.0% (1/102) and 2.6% (2/78) of the 2020 and 2022 CDSs, respectively. The mutations c.7 A>G and c.1164 C>T were detected in all 3 years; a statistically significant difference (*χ*^2^ = 11.256, *P* < 0.05) was identified in the detection rate of the c.7 A>G mutation between 2020 (9.8%, 10/102), 2021 (1.1%, 1/92), and 2022 (1.3%, 1/78). In addition, differences in the detection of the c.1164 C>T mutation in the 2020 and 2021 sequences (16.7%, 17/102 and 3.3%, 7/92, respectively) were also statistically significant (*χ*^2^ = 9.833, *P* < 0.05) (Additional file 6).

### The types of multiple mutations in the CDSs and their evolutionary relationships

Upon alignment with the reference sequence (ID: XM_001613407.1), a total of 12 haplotypes were identified among the 272 complete *pvcrt-o* gene CDSs (Table 4), with a He measured at 0.279. Of these 12, ten haplotypes were found in the CDSs from 2020, eight were from the 2021 sequences, and six were from 2022 (Table 4), with He values of 0.4327, 0.3530, and 0.4404, respectively. From this, it is clear that the number of haplotypes decreased from year to year. Hap_1 occurred with the highest frequency overall, accounting for 75.7% (206/272) of the total haplotypes identified. Furthermore, it was determined to be completely identical to the reference sequence (ID: NC_009906.1), indicating its classification as wild type. The remaining 11 haplotypes(Hap_2-Hap_12) were mutant sequences that included five single mutants, two double mutants, one triple mutant formed by the simultaneous insertion of three linked base of “28th-AAG-30th” (producing the “K10 insertion” mutation), and three quadruple mutants formed by accumulating one additional base substitution on this basis (Table 4). The proportions of these four variants (Hap_3, Hap_5, Hap_9, Hap_10) were 8.8% (24/272), 1.1% (3/272), 1.5% (4/272), and 0.7% (2/272), respectively.

**Table 4:** Structural polymorphism of CQ resistance molecular marker *pvcrt-o* gene in *P. vivax* isolates collected from Yunnan Province’ vivax malaria cases from 2020 to 2022

Order	Haplotypes	Multiplicity structure	No. of all CDSs (n = 272) No. (F, %)	Haplotype frequency between years			No. of all (n = 65) No. (F, %)	Frequency of promoter deletion		
				2020 (n = 102) No. (F, %)	2021 (n = 92) No. (F, %)	2022 (n = 78) No. (F, %)		2020 (n = 23) No. (F, %)	2021 (n = 24) No. (F, %)	2022 (n = 18) No. (F, %)
1	Hap_1	0	206 (75.7)	76 (27.9)	73 (26.8)	57 (21.0)	59 (21.7%)	20 (7.4)	21 (7.7)	18 (6.6)
2	Hap_2	S388S	15 (5.5)	7 (2.6)	2 (0.7)	6 (2.2)	3 (1.1%)	2 (0.7)	1 (0.4)	0
3	Hap_6	P13S	2 (0.7)	–	1 (0.4)	1 (0.4)	0	0	0	0
4	Hap_8	C258S	2 (0.7)	1 (0.4)	1 (0.4)	0	0	0	0	0
5	Hap_11	I3V	2 (0.7)	2 (0.7)	0	0	0	0	0	0
6	Hap_12	C312C	1 (0.4)	1 (0.4)	0	0	0	0	0	0
7	Hap_4	I3V/S388S	10 (3.7)	8 (2.9)	1 (0.4)	1 (0.4)	1 (0.4)	1 (0.4)	0	0
8	Hap_7	C258S/C312C	1 (0.4)	0	1 (0.4)	0	0	0	0	0
9	Hap_3	InsK10	24 (8.8)	1 (0.4)	12 (4.4)	11 (4.0)	2 (0.7)	0	2 (0.7)	0
10	Hap_5	InsK10/D329D	3 (1.1)	1 (0.4)	0	2 (0.7)	0	0	0	0
11	Hap_9	InsK10/T344T	4 (1.5)	3 (1.1)	1 (0.4)	0	0	0	0	0
12	Hap_10	InsK10/S388S	2 (0.7)	2 (0.7)	0	0	0	0	0	0

F: frequency; InsK10: upon alignment with the reference sequence, three base insertions of “AAG” were observed at the 28th–30th base pair of the CDS sequence, resulting in a triple mutation

**Fig. 2:**
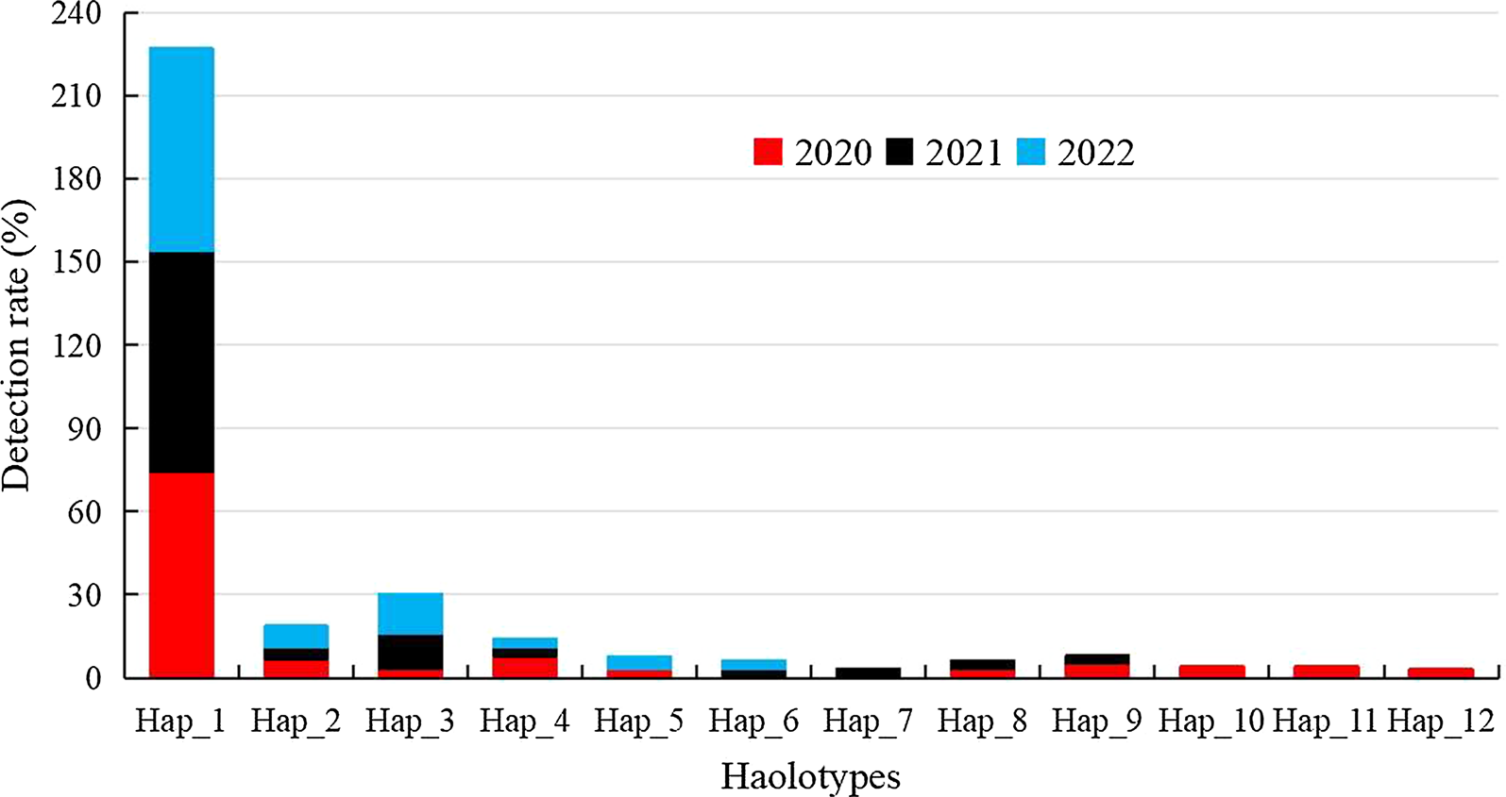
The distribution of detected 12 haplotypes between different years in Yunnan Province

These haplotypes including Hap_1, Hap_2, Hap_3, and Hap_4 were detected in CDSs from 2020, 2021 and 2022, accounting for 93.8% (255/272) of the total. The detection rate of mutant haplotype Hap_4 decreased from 7.8% (8/102) in 2020 to 1.1% (1/92) in 2021 and 1.3% (1/78) in 2022, a statistically significant decrease (*χ*^2^ = 9.922, *P* < 0.05). In addition, the detection rate of the “K10 insertion” haplotype Hap_3 increased from 1.0% (1/102) in 2020 to 13.0% (12/92) in 2021 and 14.1% (11/78) in 2022, with a difference that was also statistically significant (*χ*^2^ = 9.530, *P* < 0.05). The remaining eight haplotypes were either represented by only 1–4 CDSs or were only detected in samples from specific years (Table 4; Fig. 2).

**Fig. 3:**
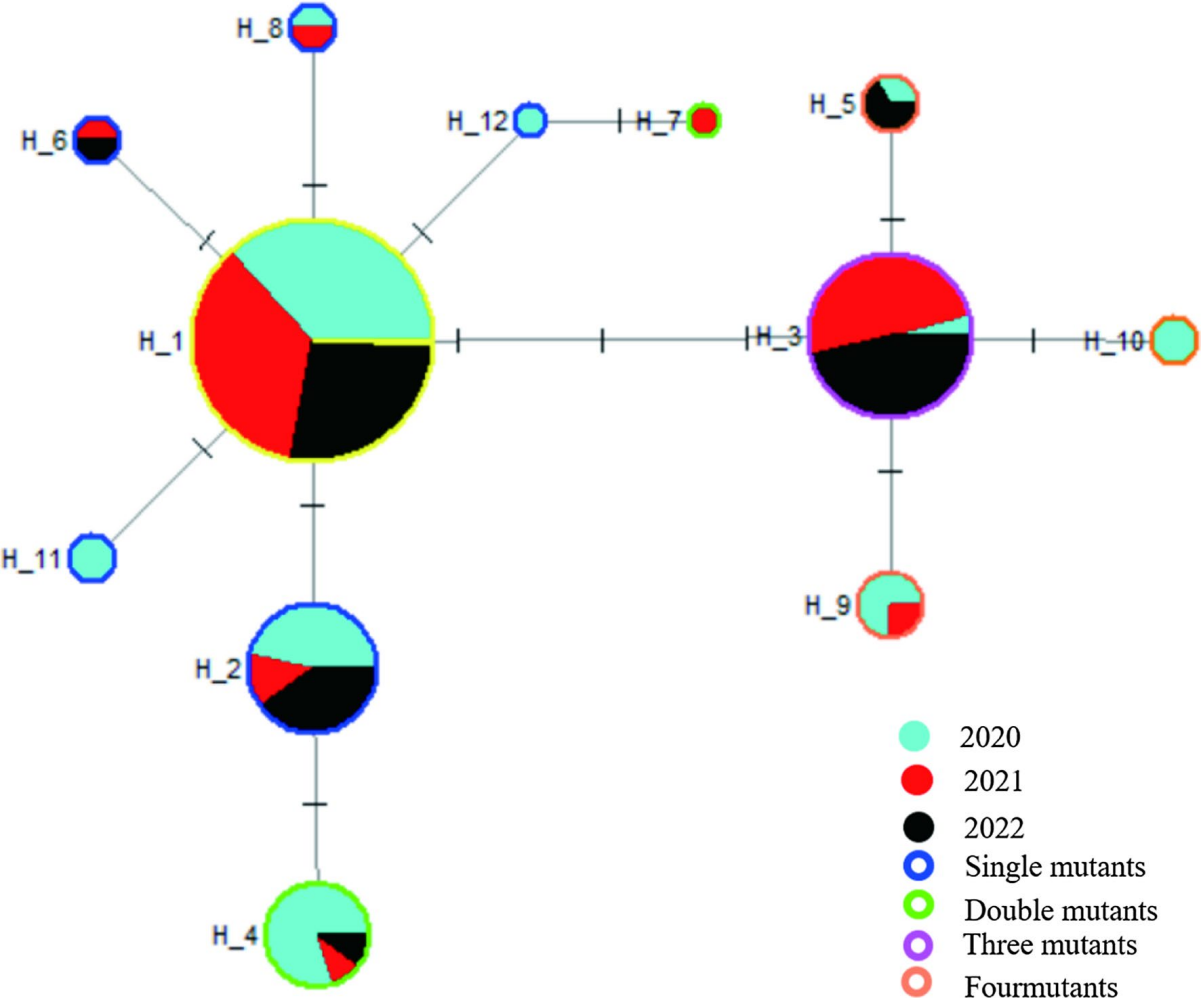
Haplotype network diagram of the *pvcrt-o* gene CDSs in *P. vivax* isolates from Yunnan Province’s vivax malaria cases (1) The size of the circle is proportional to the number of isolates showing a particular haplotype; (2) lines represent the evolutionary steps connecting haplotypes

The intermediate network diagram of the 12 haplotypes reveals a gradual evolution of the mutant haplotypes (Hap_2 to Hap_12) from the wild type haplotype (Hap_1) in multiple directions (Fig. 3). Specifically, the haplotypes Hap_3, Hap_5, Hap_9, and Hap_10, which contain the insertion of three linked bases (“28th-AAG-30th “), form branches representing evolution beyond three levels. Notably, the tertiary evolutionary haplotype (Hap_3) was predominantly observed in the sequences from 2021 to 2022, accounting for 95.8% (23/24) of the total mutations (Fig. 3). On the other hand, the primary evolutionary haplotypes Hap_2, Hap_8, Hap_11, Hap_12, and some secondary evolutionary haplotypes, such as Hap_4, dominated the 2020 CDSs (80.0%, 8/10).

### Promoter deletion linked to *pvcrt-o* gene

Among the sequences of obtained 272 CDSs, 23.8% (65/272) of the samples exhibited a deletion of 14 base pairs in the promoter region. The deleted sequence was “630th-TCACGTTATCTGCA-643th”. The distribution of these deletions across the years 2020, 2021, and 2022 was 35.4% (23/65), 36.9% (24/65), and 27.7% (18/65), respectively (Table 4). However, the differences in the detection of promoter region deletions among the samples from different years were not statistically significant and occurred in the following proportions: 22.5% (23/102) in 2020, 26.1% (24/92) in 2021, and 23.1% (18/78) in 2022 (*χ*^2^ = 0.373, *P* > 0.05).

The highest proportion of promoter region deletions was observed in these samples within the identified wild type haplotype (Hap_1) CDSs, accounting for 28.6% (59/206) of the total. In the years 2020, 2021, and 2022, the proportions were 33.9% (20/59), 35.6% (21/59), and 30.5% (18/59), respectively. Additionally, a few samples with mutations in haplotypes Hap_2, Hap_3, and Hap_4 also exhibited promoter region deletions, but only in the samples from 2020 to 2021 (Table 4). Two samples were also marked by promoter deletions combined with K10 insertions, both of which were from the samples in 2021 (Table 4).

## Discussion

The *pvcrt-o* gene is situated at both the approximate two-fifth mark and the nearly central region of chromosome 1 in *P. vivax*, specifically between positions 330,260–334,540 of 830,022 (link to NCBI). Spanning a total length of 4281 bp, it comprises 14 exons and 13 introns, with the capacity to encode 424 amino acids [41, 53]. In contrast, the *pfcrt* gene in *P. falciparum*, which is also associated with CQ resistance, likewise encodes a 424 amino acid chain despite having a shorter gene length (3096 bp) and a gene structure containing only 13 exons and 12 introns (https://www.ncbi.nlm.nih.gov/genome/gdv/browser/gene/?id=2655199) [41, 44]. These differences aside, the CQ-resistant transport protein (CRT) of both *Plasmodium* species has ten transmembrane fragments [41].

The detection of a small number of SNP sites in the *pvcrt-o* gene CDSs in this study is consistent with the findings of other researchers, but with a predominance of unreported types [54–56]. Among the seven SNPs detected in this study, three were nonsynonymous mutations (I3V, P13S, and C258S). Although they were found in the same exon 1–7 region as nonsynonymous mutations identified in *pvcrt-o* genes from isolates in the Brazilian Amazon region and China-Myanmar border area [48, 57], the specific mutation sites and amino acid substitutions (including L47S, L242P, S249P [54], and T2I, R19C, N57H [55]) in those studies differed from these findings. Additionally, the identified four synonymous mutations, including C312C, D329D, T344T, and S388S, were located in the exon 9–14 region near the 3′-end of the *pvcrt-o* gene, where previously detected synonymous mutations had not been localized. The four synonymous mutations (L122L, P565P, Q782Q and K807K) detected by Ganguly et al. in the West Bengal, India isolate [53], for instance, were limited to the exon 1–7 region only.

It is worth considering whether these differences are related to the different length of the DNA sequences analyzed in everyone’s studies, or whether they are impacted by differences in the reference sequences used for SNP identification. Regardless, it should be noted that the CDS of the *pvcrt-o* gene was meticulously compared with the mRNA reference sequence (ID: XM_001613407.1) in this study, and each SNP was confirmed through careful inspection of the “.ab1” trace files. Hence, the identified SNP types can be considered reliable. Additionally, the repeated detection of the previously reported I3V mutation provides further evidence for the correct identification of SNPs in this study [56].

The detected three non-synonymous mutations—I3V, P13S, and C258S—were all situated near the N-terminus of *Pv*CRT, but only the C258S variant appeared in the 7th TMD of *Pv*CRT (Additional file 3). The synonymous mutations D329D, T344T, and S388S were found in the 8th, 9th, and 10th TMDs of *Pv*CRT. There is still no historical information available as whether these variants have negative effects on *Pv*CRT as those caused by mutations in the 72th–76th amino acid coding region [44, 45]. Other researchers have identified non-synonymous mutations T2I, R19C, Q34H, P38L, L47S, and N57H [41], L384F and Y390C [54, 55] in the N-terminus of *Pv*CRT (in the 10th TMD), and synonymous mutations L242P and S249P [54, 55] in the 7th TMD, raising speculation about the occurrence of functional alterations in *Pv*CRT.

The “K10 insertion” mutation in the *pvcrt-o* gene has previously been associated with CQ resistance in *P. vivax* [49]. In this study, the “K10 insertion” was detected in 12.1% (33/272) of the isolates, which is lower than the detection rates reported by other researchers based on samples from the China-Myanmar border region (which ranged from 19.0 to 64.1%) [53, 56, 58, 59], Myanmar (48.3–72.7%) [60], and Thailand (76%) [51]. This distinction highlights the heterogeneity in the temporal and spatial distribution of the “K10 insertion” mutation in the Greater Mekong Subregion (GMS) [61]. However, it was also observed that the “K10 insertion” has appeared with increasing frequency over the years. In addition, fourfold mutations accumulated on the basis of the “K10 insertion,“ resulted in the detection of three haplotypes (Hap_5, Hap_9, Hap_10), accounting for 3.3% (9/272) of the samples. This is a higher proportion than 1.5% (3/199) which was reported by Wang *et al*., based on samples from the China-Myanmar border region in 2012. Moreover, the Ka/Ks ratio of the *P. vivax* population in this study reached 3.4501, indicating an ongoing positive selection for mutation types in the *pvcrt-o* gene of *P. vivax* due to environmental factors. This suggests that Yunnan Province may have to pay attention to the impact of mutant strains when using CQ to treating imported *P. vivax* infections.

The role of promoters in gene expression regulation primarily involves their influence on the binding of RNA polymerase to the template strand. Research has shown that the resistance of *P. vivax* populations in Africa to anti-malarial drugs is associated with the promoter mutations in the gene that inhibit *Pv*CRT expression [62]. In this study, the detected multiple base deletion in promoter region of the *pvcrt-o* gene differs in length and position from previous reports. The 14 bp deletion sequence (“630th-TCACGTTATCTGCA-643th”/707) was identified near the 3′-end, whereas Silva et al. found a 19 bp deletion sequence (“235th-CGAAATTTGAGAAATTCTG-253th”/707) in the middle region of the promoter in populations from the Brazilian Amazon region [63]. Therefore, it may be necessary to evaluate potential different effects of the two partial deletions sequence in promoter regions so as to alert the resistant degree of *P. vivax* to CQ in different vivax malaria endemic areas. Furthermore, the current promoter detection rate in *P. vivax* isolates from the Yunnan Province is only 23.8% (65/272), far lower than the 47.6% (11/23) reported in Brazil. Therefore, continued monitoring is essential to gauge the possibility of an expansion in the prevalence of promoter deletions.

This study not only established a practical first generation sequencing method for *pvcrt-o* but also, for the first time, obtained a batch of the *pvcrt-o* gene CDSs in *P. vivax* strains from vivax malaria cases in Yunnan Province, further enriching the shared data in GenBank. Nonetheless, some limitations of the present research have been identified. Firstly, the unique structure of the *pvcrt-o* gene in *P. vivax* (such as the widespread distribution of Poly T) has posed challenges for PCR amplification and the sequencing of all relevant fragments and their products. As a result, obtaining both the full CDSs and full promoter sequence was only successful 89.5% (272/304) of the time. There is the disadvantage to not able simultaneously obtain both full *pvcrt-o* gene and promoter DNA sequences from every case’ samples, which might partially reduce the objectivity as them to indicate the *P. vivax* clinical strains’ sensitivity to CQ. Secondly, due to limited access to shared data, this paper lacks sufficient comparisons for interpreting some of the study’s findings. In future research, the experimental protocol for *pvcrt-o* first generation sequencing needs to be optimized to improve the acquisition rate of target sequences, enabling effective implementation of the surveillance protocol outlined in the WHO’s *Malaria Surveillance, Monitoring & Evaluation: a Reference Manual* (2018) [36].

## Conclusion

The identified mutations in the *pvcrt-o* gene in *P. vivax* strains collected from vivax malaria cases from Yunnan Province has been low accumulate in recent years and has involved a limited malaria patients. Notably, this study detected no mutations in the amino acid coding region 72–76, which is strongly associated with CQ resistance. However, a certain proportion of “K10 insertions” and partial sequence deletions in the gene promoter region were identified. Therefore, further investigation is needed to determine whether these mutations have an impact on the normal expression of *Pv*CRT inside *P. vivax* strains, as this could have a significant impact on the treatment course of cases in Yunnan Province and beyond.

### Supplementary Information

**Additional file 1.** Confirmation of malaria case infected with mono-*Plasmodium vivax* in Yunnan Province.

**Additional file 2.** Detailed annotation of PCR amplification region of *pvcrt-o* gene in *P. vivax* strains based on using 3 pairs primers.

**Additional file 3.** The predicted 3D structural diagram of *P. vivax* chloroquine resistance transporter protein (*Pv*CRT).

**Additional file 4.** The distribution of vivax malaria cases diagnosed by Yunnan Province in different years and prefectures.

**Additional file 5.** The true base substitutions at seven loci confirmed by checking sequencing peaks in ‘.ab1’ file.

**Additional file 6.** The detection rate change of seven base substitution between different years.

## Data Availability

Not applicable.

## Abbreviations

*pvcrt-o*: *Plasmodium vivax* chloroquine resistance transporter protein orthologous gene
CDS: Coding DNA sequences
MAF: Minor allele frequency
CQ: Chloroquine
WHO: World Health Organization
*pvmdr1*: *P. vivax* multi-drug resistant gene
*pfcrt*: *P. falciparum* chloroquine resistance transport protein gene
*Pf*CRT: *P. falciparum* chloroquine resistance transport protein
YPMDRL: Yunnan Province Malaria Diagnosis Referent Laboratory
DBS: Dried blood spots
SNP: Single nucleotide polymorphism
π: Nucleotide diversity
He: Heterozygosity
*Pv*CRT: *P. vivax* chloroquine resistance transporter protein
*χ*^2^: Chi-square test
CRT: CQ resistant transport protein
GMS: The Greater Mekong Subregion
F: Frequency
P: Proportion
