# Supplementary material for: Molecular surveillance of chloroquine resistance in Plasmodium vivax isolates from malaria cases in Yunnan Province of China using pvcrt-o gene polymorphisms
Source: Malar J. 2023 Nov 8;22:338. doi: 10.1186/s12936-023-04776-z (PMC10631137; doi:10.1186/s12936-023-04776-z)
Supplement: Supplementary file 2 — Additional file 2. Detailed annotation of PCR amplification region of pvcrt-o gene in P. vivax strains based on using 3 pairs primers. [file 12936_2023_4776_MOESM2_ESM.docx]

**Additional file 2**

**Detailed annotation of PCR amplification region of *pvcrt-o* gene in *P. vivax* strains based on using 3 pairs primers**

**
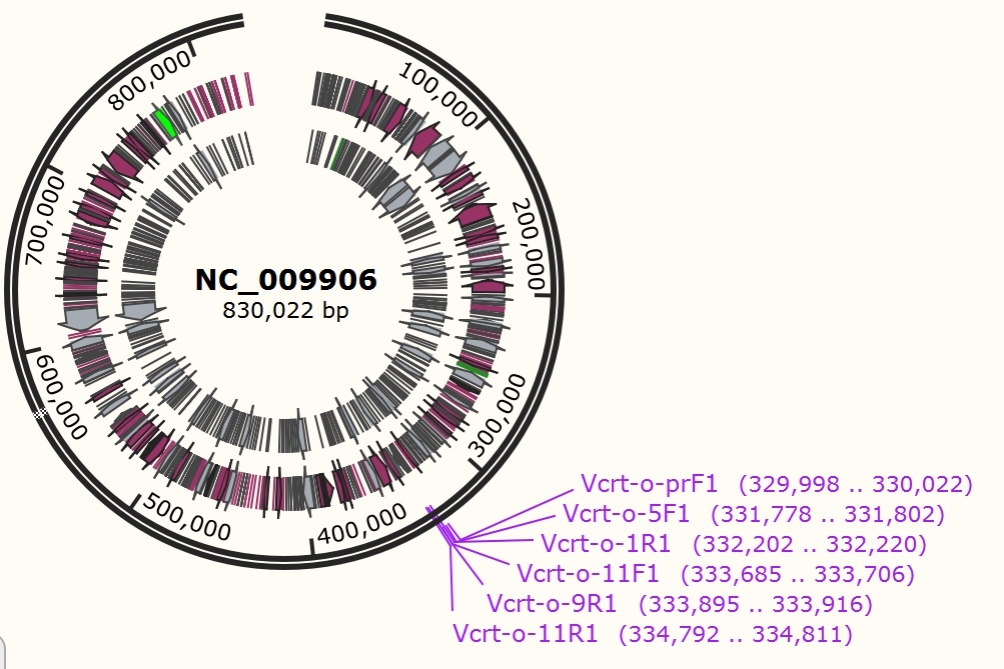
**

**Fig. S1 The primers were located to referent sequence NC_009906 by using SnapGene 4.1.9 software**


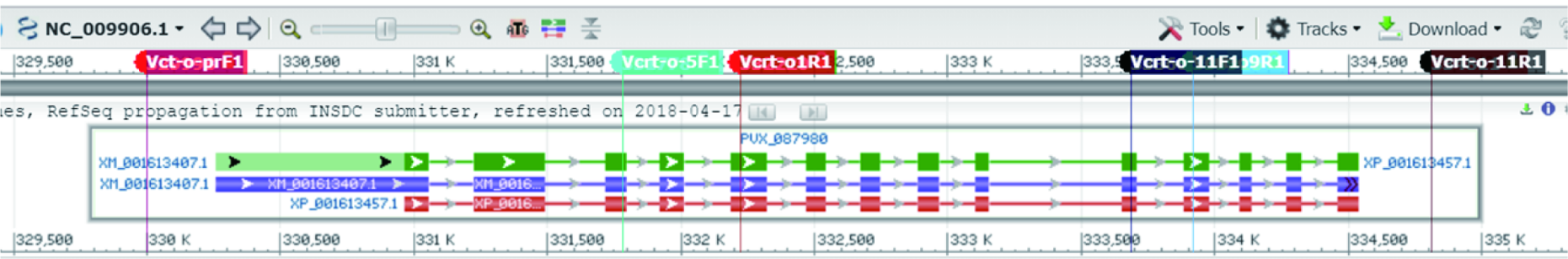


**Fig. S2 Marked amplificative interval of *pvcrt-o* gene based on three pairs primers at NCBI website database**
