# Supplementary material for: Molecular surveillance of chloroquine resistance in Plasmodium vivax isolates from malaria cases in Yunnan Province of China using pvcrt-o gene polymorphisms
Source: Malar J. 2023 Nov 8;22:338. doi: 10.1186/s12936-023-04776-z (PMC10631137; doi:10.1186/s12936-023-04776-z)
Supplement: Supplementary file 3 — Additional file 3. The predicted 3D structural diagram of P. vivax chloroquine resistance transporter protein (PvCRT). [file 12936_2023_4776_MOESM3_ESM.docx]

**Additional file 3**

**The predicted 3D structural diagram of *P. vivax* chloroquine resistance transporter protein (*Pv*CRT)**


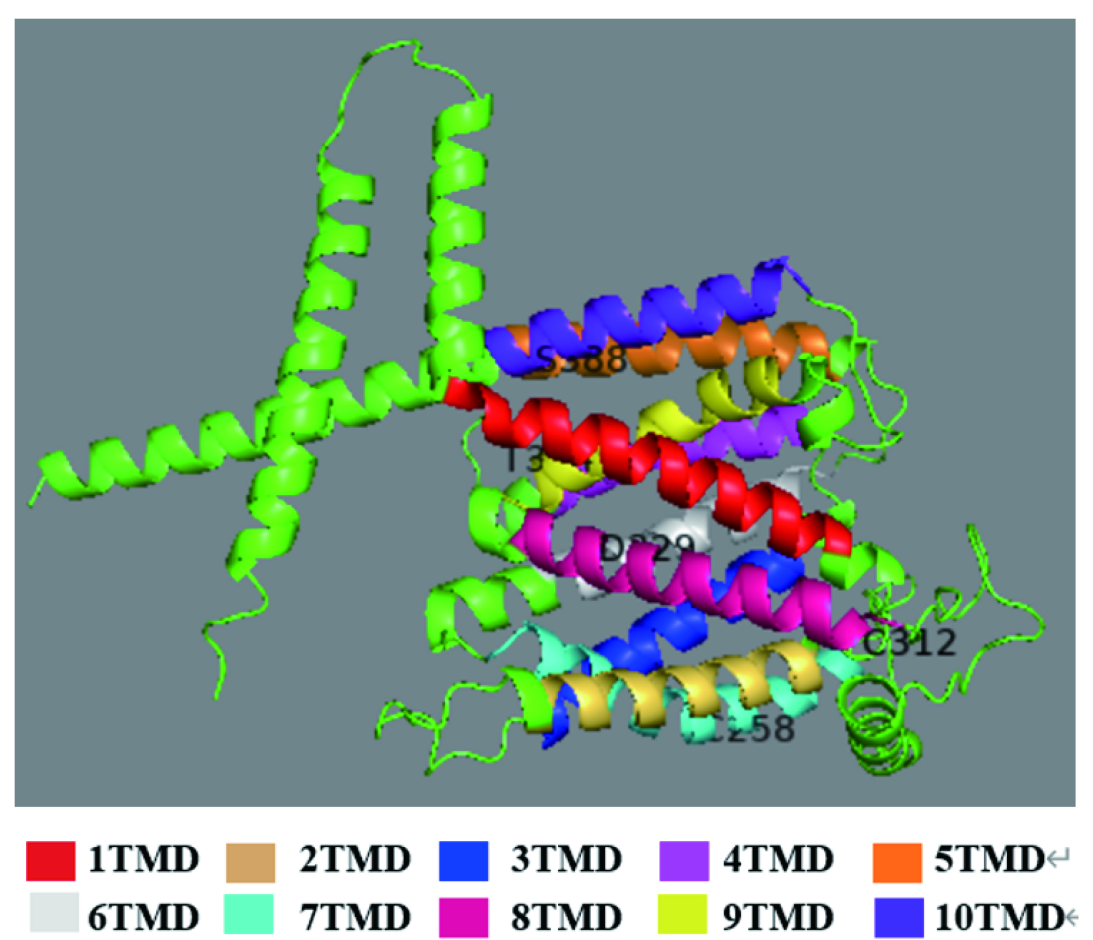


**Fig. S1 Detailed annotation of 10 TMDs in *Pv*CRT amino acid chain (GenBank ID: XP_001613457.1) from *Plasmodium vivax* Sal-I strain.** (1) The spatial structure of the *Pv*CRT amino acid chain (GenBank ID: XP_001613457.1) was predicted using SWISS-MODEL online software（https://swissmodel.expasy.org/interactive）as Model Q9N623.1. A, which these amino acids from 5^th^ aa to 424^th^ aa (424 amino acids in total length) enters the model; (2) The ten TMDs of *Pv*CRT highlighted by using different color; (3) The 6 amino acids labeled in *Pv*CRT were these amino acids affected by the SNPs in this research.
