# Supplementary material for: Molecular surveillance of chloroquine resistance in Plasmodium vivax isolates from malaria cases in Yunnan Province of China using pvcrt-o gene polymorphisms
Source: Malar J. 2023 Nov 8;22:338. doi: 10.1186/s12936-023-04776-z (PMC10631137; doi:10.1186/s12936-023-04776-z)
Supplement: Supplementary file 4 — Additional file 4. The distribution of vivax malaria cases diagnosed by Yunnan Province in different years and prefectures. [file 12936_2023_4776_MOESM4_ESM.docx]

**Additional file 4**

**The distribution of vivax malaria cases diagnosed by Yunnan Province in different years and prefectures**

| **Table S1 The prefecture distribution of 375 vivax malaria cases diagnosed in Yunnan Province from January 2020 to December 2022** | | | | |
| --- | --- | --- | --- | --- |
| **Prefectures of diagnosis** | **Total**  **No. (P, %)** | **2020**  **No. (P, %)** | **2021**  **No. (P, %)** | **2022**  **No. (P, %)** |
| **Total** | 375 (100.0) | 154 (41.1) | 126 (33.6) | 95 (25.3) |
| **Dehong** | 274 (73.1) | 107 (39.1) | 89 (32.5) | 78 (28.5) |
| **Baoshan** | 45 (12.0) | 23 (51.1) | 16 (35.6) | 6 (13.3) |
| **Lincang** | 32 (8.5) | 14 (43.8) | 11 (34.3) | 7 (21.9) |
| **Kunming** | 12 (3.2) | 6 (50.0) | 4 (33.3) | 2 (16.7) |
| **Dali** | 4 (1.1) | 0 | 3 (75.0) | 1 (25.0) |
| **Xishuanbanna** | 2 (0.5) | 0 | 1 (50.0) | 1 (50.0) |
| **Honghe** | 4 (1.1) | 2 (50.0) | 2 (50.0) | 0 |
| **Qujing** | 1 (0.3) | 1 (100.0) | 0 | 0 |
| **Chuxiong** | 1 (0.3) | 1 (100.0) | 0 | 0 |

Note: P is proportion.
