# Supplementary material for: Molecular surveillance of chloroquine resistance in Plasmodium vivax isolates from malaria cases in Yunnan Province of China using pvcrt-o gene polymorphisms
Source: Malar J. 2023 Nov 8;22:338. doi: 10.1186/s12936-023-04776-z (PMC10631137; doi:10.1186/s12936-023-04776-z)

**Additional file 5**

**The true base substitutions at seven loci confirmed by checking sequencing peaks in ‘.ab1’ file.**

**Confirmation of the base substitute from wildtype to mutant type at 7 loci in *pvcrt-o* gene CDSs from *P. vivax* strains based on checking sequencing peaks. All checking as following:**


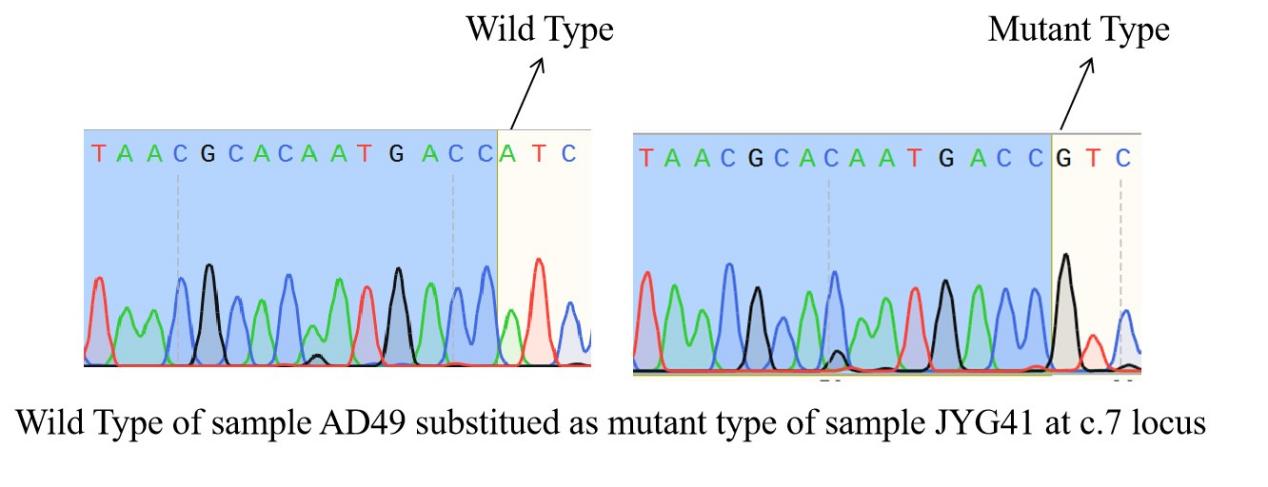

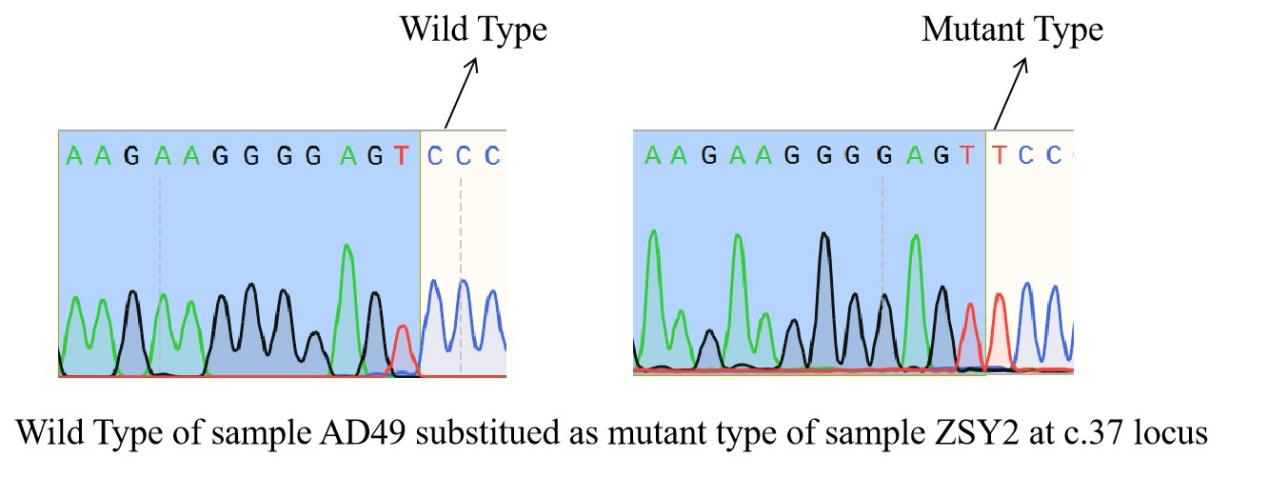

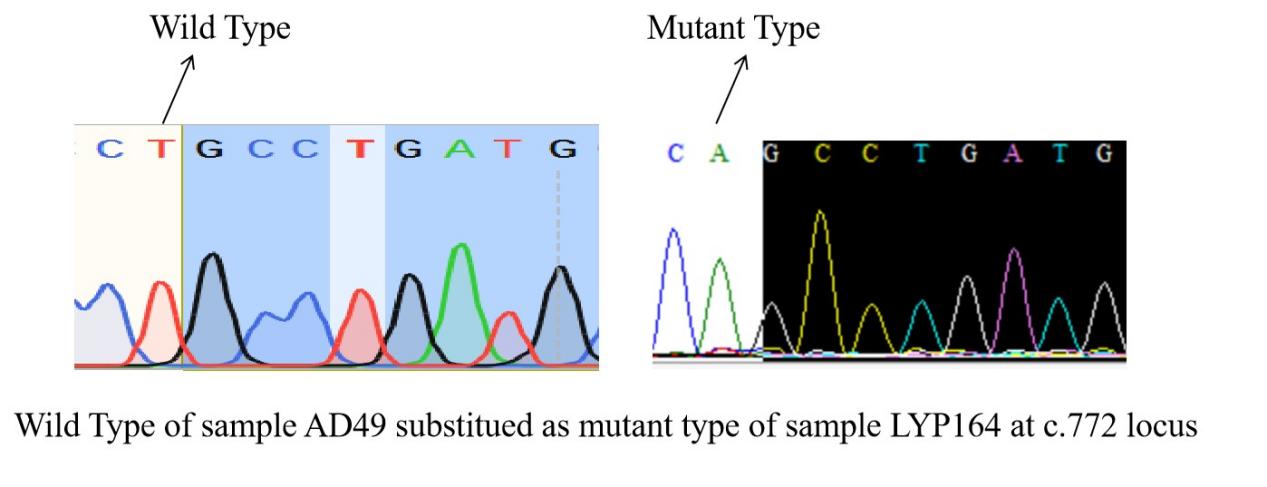

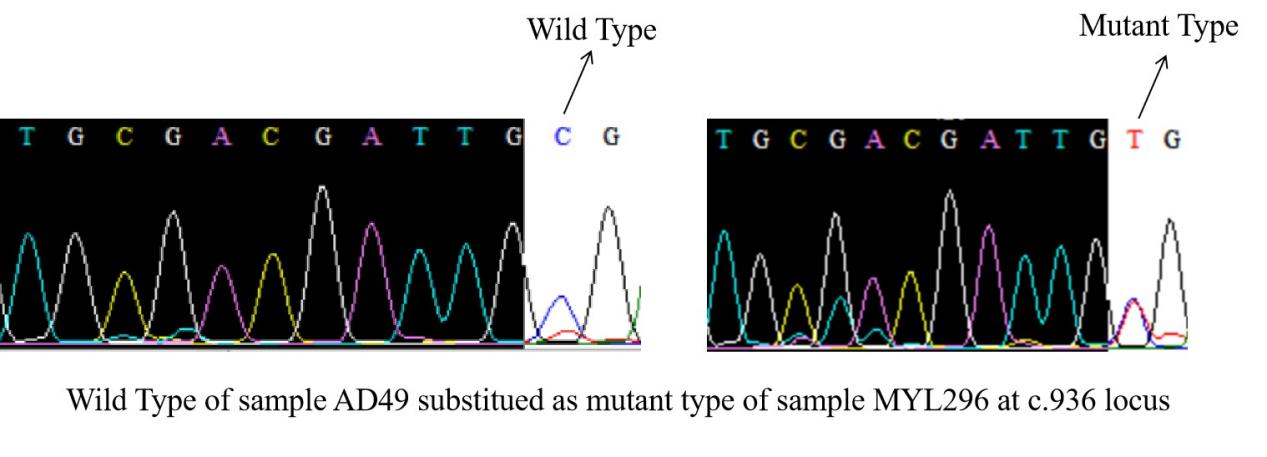

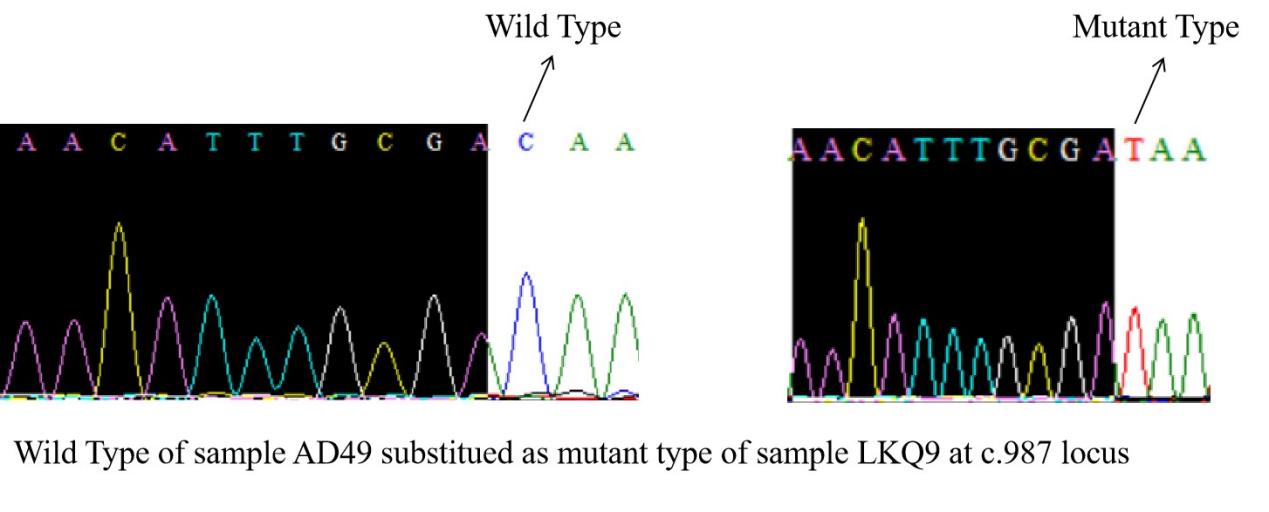

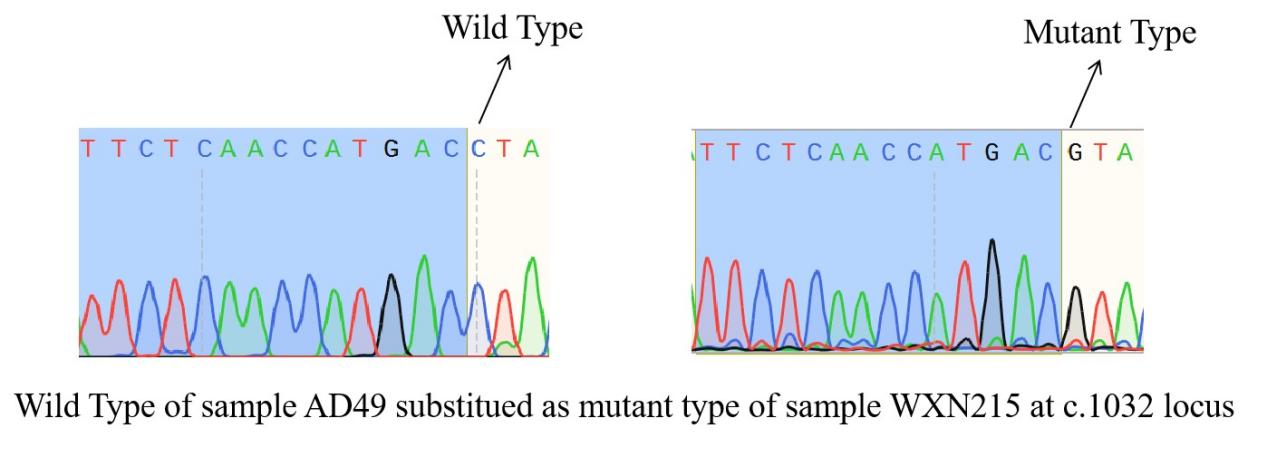

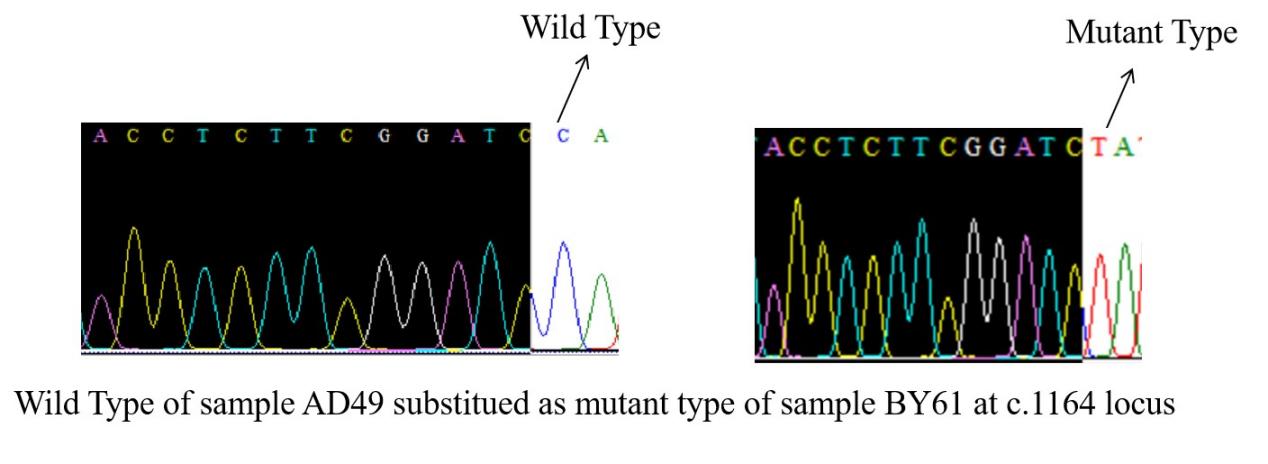

Supplement: Supplementary file 5 — Additional file 5. The true base substitutions at seven loci confirmed by checking sequencing peaks in ‘.ab1’ file. [file 12936_2023_4776_MOESM5_ESM.docx]
