# Supplementary material for: Molecular surveillance of chloroquine resistance in Plasmodium vivax isolates from malaria cases in Yunnan Province of China using pvcrt-o gene polymorphisms
Source: Malar J. 2023 Nov 8;22:338. doi: 10.1186/s12936-023-04776-z (PMC10631137; doi:10.1186/s12936-023-04776-z)
Supplement: Supplementary file 6 — Additional file 6. The detection rate change of seven base substitution between different years. [file 12936_2023_4776_MOESM6_ESM.docx]

**Additional file 6**

**The detection rate change of seven base substitution between different years.**


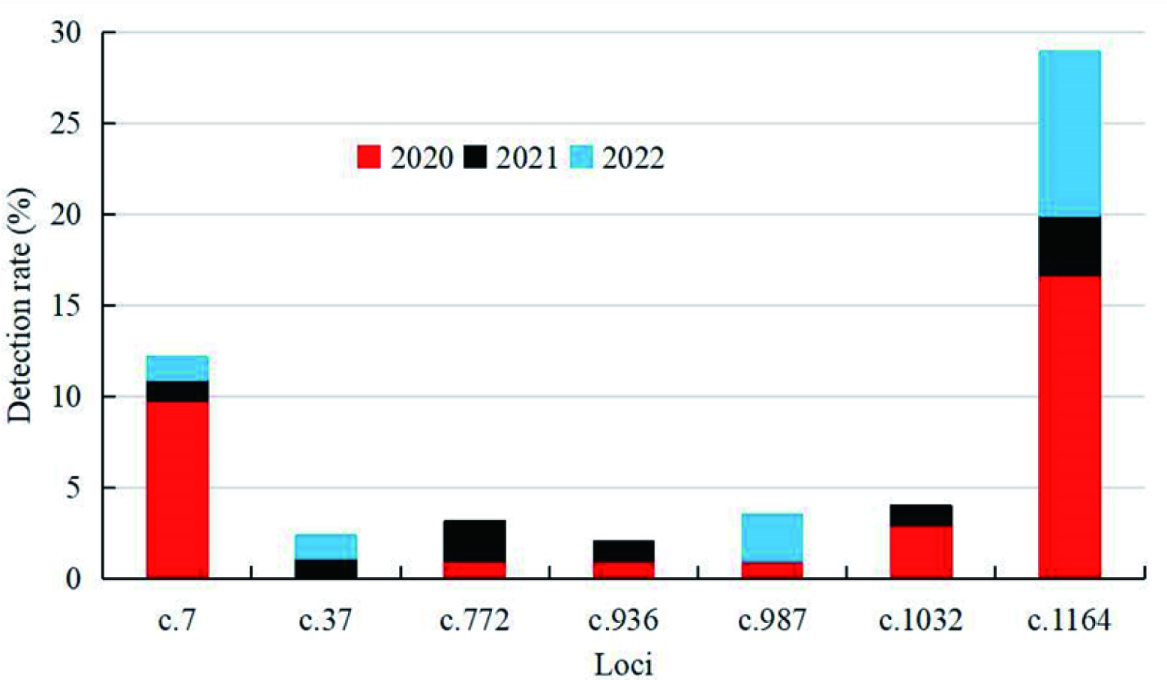


**Fig. S1** **The change of detection rate of seven SNPs between different years**
